# Stable Soil Moisture Improves the Water Use Efficiency of Maize by Alleviating Short-Term Soil Water Stress

**DOI:** 10.3389/fpls.2022.833041

**Published:** 2022-04-18

**Authors:** Li Niu, Zhuan Wang, Guolong Zhu, Kefan Yu, Ge Li, Huaiyu Long

**Affiliations:** ^1^Institute of Agricultural Resources and Regional Planning, Chinese Academy of Agricultural Sciences, Beijing, China; ^2^Beijing Liangxiang Lanxin Hydraulic Engineering & Design Co., Ltd, Beijing, China

**Keywords:** temporal variation of soil moisture, negative pressure irrigation, crop initiate drawing water, maize, physiological response

## Abstract

Weaker temporal variation of soil moisture can improve crop water use efficiency (WUE), but its physiological mechanism was still unclear. To explore the mechanism, an organized experiment was conducted in Beijing from June to September. From the jointing stage to maturity stage of maize, stable soil moisture (SSM) and fluctuating soil moisture (FSM) were established with Pressure Potential Difference-Crop Initiate Drawing Water (PCI) and manual irrigation (MI), respectively, to explore the physiological mechanism of SSM to improve maize WUE. Among them, PCI treatments were set with 3 pressure differences of -5, -10, and -15 kPa, and MI treatment was watering every 3 days with the irrigation amount of 9.3 mm. The results showed that (1) after water treatment, the average soil water content of PCI-5 kPa, PCI-10 kPa, PCI-15 kPa, and MI treatments were 53% field capacity (FC), 47, 38, and 78% FC, respectively. It was SSM with weak temporal variation under PCI treatments, and FSM with medium temporal variation under MI treatment. (2) PCI treatments reduced the content of proline, malondialdehyde, and abscisic acid in each organ of maize. (3) Compared with FSM 78% FC, the maize root activity at the filling stage of 53% FC SSM and 47% FC SSM increased significantly by 57.1 and 28.6%, respectively, and the carbon isotope discrimination value (Δ^13^C) in bracts of the two treatments increased by 18.3 and 10.4%, respectively. (4) There was a very significant positive correlation between WUE based on biomass (WUE_b_) and Δ^13^C in bracts. In conclusion, a large temporal variation of soil moisture was an important factor that caused water stress in maize. Under SSM treatments, the accumulation of abscisic acid, proline, and malondialdehyde was synergistically reduced. SSM improved the WUE of maize by alleviating short-term soil water stress caused by the fluctuation of soil moisture.

## Introduction

Maize is not only the most productive food crop but also an important source of feed in China, which plays an important role in meeting human dietary needs (Klopfenstein et al., 2013; Hou et al., 2021). The water consumption of maize during the whole growth period was relatively large. Xiao’s study suggested that summer maize with a yield of 10,500–12,000 kg/hm^2^ had a water requirement of 3500–4000 m^3^ (Xiao et al., 2008). At present, water shortage is a worldwide problem (Zhang Y. et al., 2019). Therefore, it is of great significance for water conservation to study the water-saving irrigation technology of maize and its water-saving mechanism.

Previous studies on the relationship between soil moisture and crops generally believed that crops have compensation effect of water deficit (Shan and Xu, 1991) and root–shoot communication mechanisms (Kang et al., 2007). Based on the two mechanisms, crops will undergo a series of physiological and biochemical reactions to avoid the harm of soil water stress, thereby improving crop yields and water use efficiency (WUE). The field trials results revealed that compared with full irrigation, maize had the highest WUE when 20–30% water deficit was applied (Liu et al., 2017). Plants respond to water deficits by regulating endogenous hormones, osmotic regulating substances, and membrane lipid peroxidation products, thereby improving WUE. Proline functions as an osmotic regulating substance, the hydrophilic protective substance of enzymes and cell structure, and free radical scavenger to protect cells under stress conditions (Naeem et al., 2018). In the case of water deficit, proline gets accumulated in plants to reduce water potential and avoid the harmful effects of water stress (Maggio et al., 2002). In response to water stress, excessive accumulation of reactive oxygen species will oxidize polyunsaturated fatty acids in membrane lipids and produce malondialdehyde (MDA) in plants. The chain polymerization of MDA with membrane proteins leads to the increase of membrane permeability and the destruction of the membrane system (Tayyab et al., 2020), which leads to the damage of ion channels, membrane proteins, and related enzymes. Therefore, MDA content can be used to evaluate the degree of lipid damage (Ma et al., 2015). The content of MDA increases with the decrease of soil water content (100, 60, and 30% FC) (Khazaei et al., 2020). Abscisic acid (ABA) is produced by the root and transported to shoot underwater stress, which regulates the stomatal aperture of leaves (Buckley, 2019), and is regarded as an important signal substance of plants in response to soil water stress. Furthermore, the accumulation of ABA in plants can inhibit the transpiration rate, root growth rate, and grain fertility (Pilet and Saugy, 1987; Wang H. et al., 2020), thus affecting the WUE (Liao et al., 2018). In addition, salicylic acid (SA) also played an important role in responding to water deficits. Previous research revealed that SA improved the tolerance of plants to drought stress by enhancing the antioxidant protection system and increasing the content of osmotic regulators (Saheri et al., 2020; Sedaghat et al., 2020). SA levels were significantly elevated in maize seedlings with water withholding for 7 days (Guo et al., 2021) and spraying 1 mM SA in the early stage of drought can alleviate the effects of drought stress and ensure the growth of maize seedlings (Bijanzadeh et al., 2019).

Root activity not only affected the growth and development of plants, but also reflected the stress resistance of plants, and the root activity first increased and then decreased with the decrease of soil water content (Wu et al., 2017). Carbon isotope discrimination value (Δ^13^C) was used to reflect the water status of crops and soil and characterize the physiological state of crops, which have a certain relationship with plant osmotic regulation and WUE (Yang and Li, 2018). It was expected to become a simple and fast method to characterize crop WUE. The relationship between WUE and Δ^13^C had been extensively studied. In a previous study of wheat, the Δ^13^C in grains was negatively correlated with leaf instantaneous WUE, total biomass WUE, and yield WUE (Wang et al., 2013). The research of maize indicated that the Δ^13^C of leaves and grains at the filling stage was negatively correlated with the WUE based on grain yield (Yang and Li, 2018). While the research on maize genome seemed to give the reason: the carbon isotope composition, WUE, and drought sensitivity of maize are controlled by a common genome segment (Avramova et al., 2019).

In summary, the relationship between maize physiological response, WUE, and Δ^13^C based on the theory of water deficit compensation effect was mostly studied under traditional irrigation technology. The soil water content was constantly undergoing the process of gradually changing from “high” to “low” and suddenly from low to high. The time variation of soil moisture was very large, and it was difficult to accurately describe the relationship between soil moisture and crops. Batista et al. (2013) had proposed a device to stabilize soil moisture using electricity. Recently, the negative pressure irrigation technology had been identified as Pressure Potential Difference-Crop Initiate Drawing Water (PCI) technology (Long et al., 2020), which can accurately, continuously, and stably control soil water content without any energy consumption. Previous studies had indicated that PCI can effectively improve crop yield and WUE compared with traditional irrigation (Bian et al., 2018; Zhao et al., 2019; Wang Z. et al., 2020; Yang et al., 2020; Zhu et al., 2020). However, the soil moisture under PCI is in a relatively stable state, and the effect of water deficit compensation does not exist in theory. The mechanism of crops’ efficient use of water under the condition of stable soil moisture (SSM) was not clear. Physiological and gene expression responses of plants under different SSMs were compared in *Petunia* (Kim et al., 2012), while the differences in the effects of stable and fluctuating soil moisture (FSM) on plants were not compared.

In this experiment, PCI technology and manual irrigation were used to form SSM and FSM, respectively. By observing malondialdehyde, proline, plant hormone, root activity, Δ^13^C, and WUE under different treatments, the physiological response of maize to temporal variations of soil moisture was explored. It is expected to lay the foundation for revealing the physiological mechanism of SSM to improve crop WUE.

## Materials and Methods

### Study Site

The maize pot experiment was conducted from June to September, 2018, in the rain shelter house in the Chinese Academy of Agricultural Sciences (39.9°N, 116.3°E) in Beijing, China. The annual average temperature and precipitation of this site were 10–12°C and 565 mm, respectively, and it has a warm and semi-humid continental monsoon climate.

### Pressure Potential Difference-Crop Initiate Drawing Water System

**FIGURE 1:**
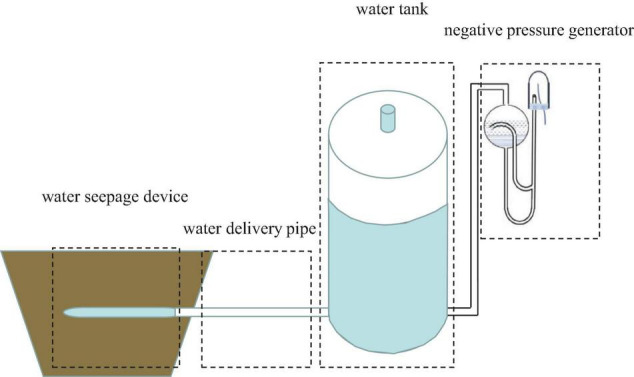
Schematic diagram of pressure potential difference-crop initiate drawing water technology device (Wang et al., 2019).

The PCI system (Patents, China ZL201610329413.3) is composed of four parts: a negative pressure generator, a water tank, a water delivery pipe, and a water seepage device (Figure 1). The heavy liquid-type negative pressure valve was used as a negative pressure generator to control the water supply pressure. The water tank was a sealed cylindrical PVC bucket (inner diameter 19 cm, height 50 cm) with a tube installed on it to observe the water level. The water delivery pipe is a transparent silicone hose connecting the water tank and the water seepage device. The water seepage device is a porous clay pipe (with an inner diameter of 1 cm, an outer diameter of 1.8 cm, and a length of 23 cm) that is water permeable and airtight. The water seepage device was buried in the middle of the potting bucket, 10 cm deep from the soil surface, and slightly inclined downward until the tail was about 1 cm lower than the head, which is conducive to the air discharge from the clay pipe. With the growth of crops and the absorption of soil moisture, the soil matrix potential decreases. The PCI system will start to irrigate when the soil matrix potential is lower than the negative pressure in the clay pipe because water will always move from the high potential region (PCI system) to the low potential region (soil). Here, the water level of the tank drops, and the air volume above the tank increases, thereby reducing the pressure in the tank and allowing the outside air to enter the tank through the silicone tube. Through this process, the dynamic balance of soil matrix potential and irrigation pressure is realized by the PCI system, thereby maintaining the soil moisture stability (Figure 1).

### Pots and Soil

Considering the specifications of the clay pipe and the uniformity of soil moisture distribution throughout the pot, pots with length, width, and height of 42, 26, and 25 cm were used for the experiment. The clay pipe was inserted into the middle of the soil through a hole (10 cm from the soil surface) punched in the middle of one side of the pot at an angle of 5–10° to the horizontal. Each pot was filled with 25 kg of soil and the texture of which was loamy clay. The basic physical and chemical properties of the soil are shown in Table 1.

**TABLE 1:** Soil basic physical and chemical properties.

Soil texture			Field capacity (V/V)	Soil bulk density	Alkaline hydrolysis nitrogen	Available phosphorus	Available potassium	OM	EC	pH
Sand	Silt	Clay								
%	%	%	(%)	(g cm^–3^)	(mg kg^–1^)	(mg kg^–1^)	(mg kg^–1^)	(g kg^–1^)	(μs cm^–1^)	(%)
52.9	22.5	24.5	22.36	1.1	93.53	34.26	194.02	16.04	129.2	6.66

### Experimental Design

Four treatments were established in this experiment, which was PCI-5 kPa, PCI-10 kPa, and PCI-15 kPa treatment and manual irrigation treatment (MI), with four replicates per treatment. The maize cultivar “Zheng dan 958” was sown on June 25, 2018. Before sowing, each pot was applied with 2.3 g of urea, 8.5 g of superphosphate, 2.8 g of potassium sulfate, and 3 L of water. Six seeds were sown in each pot, and the seedlings were thinned at the third leaf stage, leaving one seedling per pot. The top application was applied with 4.7 g of urea per pot at the twelfth leaf stage. The water consumption of summer maize in the local field was 5.0–7.0 mm/day (Xiao et al., 2008). Considering that there was no water leakage in potting soil, an irrigation amount of 1 L was given to each pot every 2 days, equivalent to 3.1 mm/day, before the jointing stage. The PCI system was activated at the jointing stage until maturity. Then the PCI treatments were irrigated through the PCI system, and the MI treatment was surface irrigated manually every 3 days with the irrigation amount of 9.3 mm.

### Sampling and Measurements

#### Soil Moisture

The soil moisture was measured every 4 days at the period of 17:00 to 18:00 from silking stage (2 weeks after the PCI system was initiated) to maturity. Three points were evenly taken around each maize plant using a soil moisture rapid measuring instrument (SU-LB, Beijing Meng Chuang Wei Ye Technology Co., Ltd., Beijing, China) with a measuring depth of 0–6 cm.

#### Soil Moisture Stability

The temporal variation coefficient (*CV*) is calculated as follows:


(1) C⁢V=σ/m

Where σ is the SD of soil moisture observed at different times, and μ is the average value of soil moisture observed at different times. If *CV* ≤ 0.1, the soil moisture belongs to weak variations if 0.1 < *CV* < 1, it belongs to medium variation, and if *CV* ≥ 1, it belongs to strong variation (Zhu et al., 2020).

The fluctuation coefficient (δ) of soil moisture was calculated as follows:


(2) $$\delta =\Sigma \,\left[\left|\theta_{\text{i}}-\theta_{i-1}\right|/\left(\left(\theta_{\text{i}}+\theta_{i-1}\right)/2\right)\right]/\left(n-1\right)$$

Where θ*_*i*_* is the observed value of soil water content (%) at *i* moment, θ*_*i*_*_–1_ is the observed value of soil water content (%) at the last moment before *i*, and *n* is the observation times of soil water content. The fluctuation coefficient reflects the stability of soil moisture, and the smaller the value is, the more stable the soil moisture is (Zhu et al., 2020).

#### Physiological Characteristics

##### Plant Water Content and Stomatal Conductance

At the grain filling stage, the root, stem, leaf, bract, and cob were sampled and weighed immediately to obtain fresh weight. Then, the samples were heated at 105°C for 30 min and dried to constant weight at 70°C. The samples were weighed to obtain the dry weight.


Plant⁢water⁢content=⁢(fresh⁢weight-dry⁢weight)×100%/fresh⁢weight

The stomatal conductance was measured using a Li-6400 portable photosynthesis instrument (LI-COR, Lincoln, NE, United States) with an LED light source between 9:00 and 11:00 AM on a sunny day (Niu et al., 2020).

##### Malondialdehyde and Proline

Sampling was performed from 9:00 to 11:00 AM at the grain filling stage. The ear leaves of maize were sampled and immersed in liquid nitrogen immediately and then transferred to a –80°C freezer for later use. For the quantification of MDA, ear leaf (0.5 g) was determined by thiobarbituric acid method with the absorbance recorded at 450, 532, and 600 nm using a UV–vis spectrophotometer (Saheri et al., 2020). For the quantification of proline, ear leaf (0.5 g) was extracted with 5 ml of 3% sulfo SA solution, and the homogenate was heated in a boiling water bath for 10 min and then cooled. The cooled homogenate was centrifuged at 3,000 *g* for 10 min. Next, 2 ml of the supernatant was added with 2 ml of distilled water, 2 ml of glacial acetic acid, and 4 ml of 2.5% ninhydrin solution, and the mixture was put into a boiling water bath for 60 min and then cooled. The cooled mixture was added with 4 ml of toluene to extract the red substance. After standing the mixture, the toluene phase was used to measure the absorbance at a wavelength of 520 nm, and the proline content was calculated according to a standard curve (Saheri et al., 2020).

##### Abscisic Acid and Salicylic Acid

For the detection of ABA and SA, the ear leaf (50 mg) was ground with liquid nitrogen and added with 0.5 ml methanol/water/formic acid (15:4:1, V/V/V) at 4°C. The extract was vortexed for 10 min and centrifuged at 14,000 rpm for 5 min at 4°C. The supernatant was collected, and the extraction process was repeated. The combined extracts were dried with a stream of nitrogen and redissolved with 80% (v/v) methanol. The extract was ultraphoniced for 1 min and filtrated (PTFE, 0.22 μm; Anpel). The ABA and SA content were detected by an LC--ESI--MS/MS system (HPLC, Shim-pack UFLC SHIMADZUCBM30A system^1^; MS, AppliedBiosystems 6500 Triple Quadrupole^2^) (Guo et al., 2021).

##### Root Activity and Δ^13^C

The roots were taken out from the soil and washed with 0.01 mol/L phosphate-buffered saline. After washing, the roots were wiped dry and stored at 4°C for later use. The root activity of maize was measured with the triphenyl tetrazolium chloride method using 0.5 g fresh root as soon as possible (Khan et al., 2014). For the quantification of Δ^13^C, the ear leaf was dried at 80°C to constant weight, then the dried leaf was ground to a fine powder and passed through a 0.15-mm sieve. A 1 mg of the sample was determined by the combination of element analyzer (Flash 2000 HT, Thermo Fisher Scientific, Waltham, United States) and isotope mass spectrometer (Delta V advantage, Thermo Fisher Scientific, United States) (Gao et al., 2018). Four biological replicates were performed for the physiological indicators.

#### Water Consumption of Plant

The water consumption of the plant was calculated according to the theory of water balance:


(3) $$E\,T_{\text{k}}=M_{\text{k}}-\Delta \,W=M_{\text{k}}-\left(\theta \,m_{\text{k}}-\theta \,m_{k-1}\right)\times m_{\text{s}}/\rho_{\text{w}}$$

In the formula, ET_k_ is the water consumption (L) of maize in the kth period, M_k_ is the irrigation amount (L) in the kth period, ΔW is the change of soil water storage (L), θm_k_ is the mass water content of soil (%) at the kth moment, θm_k–1_ is the mass water content of soil (%) at the last moment before k, m_s_ is the mass of the soil in the pot (kg), and ρ_w_ is the density of water (1 g.cm^–3^) (Li et al., 2017).

#### Water Use Efficiency

At the maturity stage, the shoot and root of maize were put into an oven at 105°C for 30 min and then dried to constant weight at 75°C. The biomass and grain yield were measured, respectively. WUE was calculated as follows:


$$\mathrm{Biomass}\,\mathrm{water}\,\mathrm{use}\,\mathrm{efficiency}\,\left({\mathrm{WUE}}_{B}\right)=\mathrm{biomass}/\mathrm{total}\,\mathrm{water}\,\mathrm{consumption}.$$

$$\mathrm{Yield}\,\mathrm{water}\,\mathrm{use}\,\mathrm{efficiency}\,\left({\mathrm{WUE}}_{Y}\right)=\mathrm{grain}\,\mathrm{yield}/\mathrm{total}\,\mathrm{water}\,\mathrm{consumption}.$$

### Statistical Analysis

ANOVA and correlation were performed with SPSS 12.5 (SPSS, Chicago, IL, United States), and the least significant difference (LSD) method was used to compare means at *P* < 0.05.

## Results

### Soil Moisture and Its Stability

From the silking stage to the maturity stage, the average soil volume water contents of MI, PCI-5 kPa, -10 kPa, and -15 kPa were 17.40% (78% FC), 11.81% (53% FC), 10.55% (47% FC), and 8.59% (38% FC), respectively, indicating that the greater the PCI pressure was, the higher the soil moisture content became. The temporal variation coefficients of soil moisture in the MI, PCI-5 kPa, PCI-10 kPa, and PCI-15 kPa treatments were 0.113, 0.068, 0.093, and 0.121, respectively, and the fluctuation coefficients were 0.120, 0.041, 0.074, and 0.066, respectively. Considering the variation coefficient and fluctuation coefficient, the PCI treatments were SSM with weak time variation, and the MI treatment was FSM with medium time variation (Table 2 and Supplementary Figure S1).

**TABLE 2:** Soil volumetric moisture content and its stability parameters under different treatments.

Treatment	The range of soil moisture content (%)	Mean value of soil moisture content (%)	Temporal variation coefficients of soil moisture	Temporal fluctuation coefficients of soil moisture
MI	13.98 (62.5%FC) ∼ 19.96 (89.3%FC)	17.40 (78%FC)	0.113	0.120
–5 kPa	10.55 (47.2%FC) ∼ 12.89 (57.6%FC)	11.81 (53%FC)	0.068	0.041
–10 kPa	9.12 (40.8%FC) ∼ 12.23 (54.7%FC)	10.55 (47%FC)	0.093	0.074
–15 kPa	6.83 (30.5%FC) ∼ 9.88 (44.2%FC)	8.59 (38%FC)	0.121	0.066

### Physiological Characteristics

#### Proline

**FIGURE 2:**
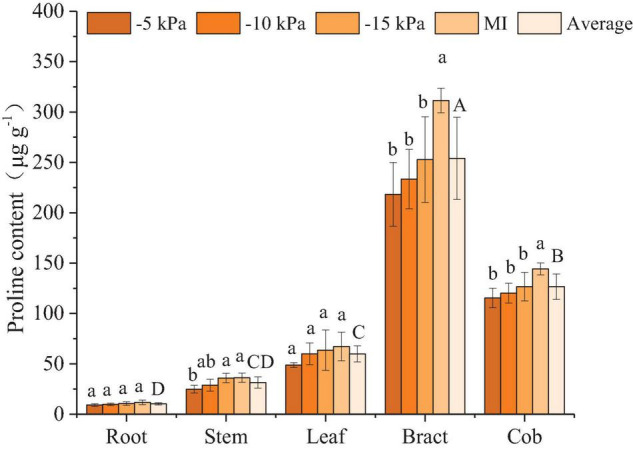
Proline content in different organs of maize under different treatments. Values represent mean ± SD. The least significant difference (LSD) method was used to test differences among treatments or organs at the *P* < 0.05 level. Different lowercase letters indicate significant differences among treatments within the same organ, and different capital letters on the average value of all treatments in each organ indicate significant differences among organs.

Compared with MI treatment, the proline content of each organ tended to decrease under PCI treatments. The stem proline content of the PCI-5 kPa treatment was significantly lower than that of the MI treatment by 31.4%, and the proline content in the bract and cob of all PCI treatments was also significantly lower than that of the MI treatment (Figure 2). The bract leaf proline contents of PCI-5 kPa, PCI-10 kPa, and PCI-15 kPa were significantly lower than MI by 29.9, 25.1, and 18.8%, respectively, and the cob proline contents of PCI-5 kPa, PCI-10 kPa, and PCI-15 kPa were significantly lower than MI by 20.0, 16.6, and 12.2%, respectively. In the range of 38% FC to 53% FC under SSM conditions, the proline content in each organ gradually increased with decreasing soil moisture content. Among them, the stem proline contents of the PCI-5 kPa and -10 kPa treatments were lower than that of the -15 kPa treatment by 30.8 and 19.9%, respectively (Figure 2). The proline content varied in different organs in the order of bract > cob > leaf > stem > root. The average proline content of bracts was 23.7, 7.1, 3.2, and 1.0 times higher than that of roots, stems, leaves, and cobs, respectively, and that of cobs was 11.3, 3.0, and 1.1 times higher than that of roots, stems, and leaves, respectively.

#### Malondialdehyde

**FIGURE 3:**
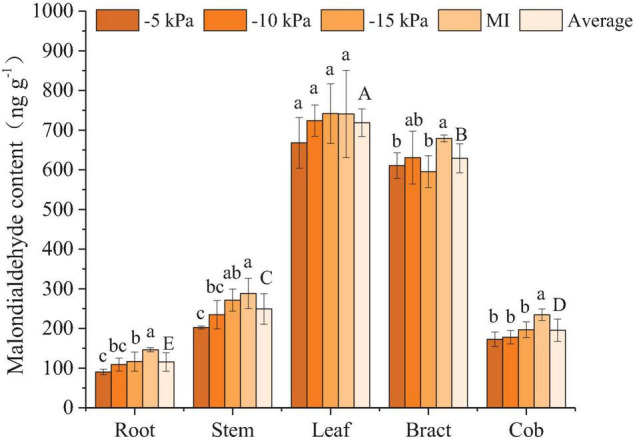
Malondialdehyde content in different organs of maize under different treatments. Values represent mean ± standard deviation. The least significant difference method was used to test differences among treatments or organs at the *P* < 0.05 level. Different lowercase letters indicate significant differences among treatments within the same organ, and different capital letters on the average value of all treatments in each organ indicate significant differences among organs.

The root MDA content of the MI treatment was significantly higher than that of the PCI-5 kPa, PCI-10 kPa, and PCI-15 kPa treatments by 38.3, 25.6, and 20.4%, respectively. Moreover, the cob MDA of MI treatment was significantly higher than PCI-5 kPa, PCI-10 kPa, and PCI-15 kPa by 26.3, 24.1, and 16.0%, respectively. Stem MDA in the MI treatment was significantly higher than that in the PCI-5 kPa and PCI-10 kPa treatments by 29.8 and 18.6%, respectively, and bract MDA in the MI treatment was significantly higher than that in the PCI-5 kPa and PCI-15 kPa treatments by 10.1 and 12.4%, respectively. Within the range of 38% FC to 53% FC under SSM conditions, the MDA content of roots, stems, leaves, and cobs increased with decreasing soil water content. Among them, the MDA content in roots and stems at 38% FC was significantly higher than that at 53% FC, while the MDA content in bracts showed a trend of first increasing and then decreasing with decreasing water supply pressure (Figure 3). The MDA content differed in different organs of maize. The average MDA content of all treatments in leaves was 0.1, 1.9, 2.7, and 5.2 times higher than that in bracts, stems, cobs, and roots, and that in bracts was 1.5, 2.2, and 4.4 times higher than that in stems, cobs, and roots (Figure 3).

#### Abscisic Acid

**FIGURE 4:**
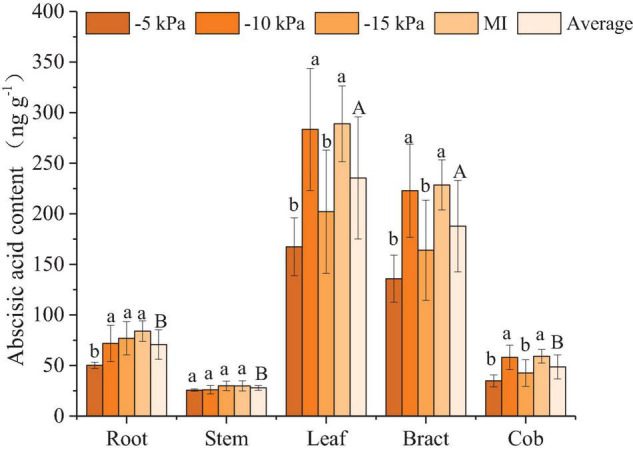
Abscisic acid content in different organs of maize under different treatments. Values represent mean ± SD. The least significant difference method was used to test differences among treatments or organs at the *P* < 0.05 level. Different lowercase letters indicate significant differences among treatments within the same organ, and different capital letters on the average value of all treatments in each organ indicate significant differences among organs.

The ABA content in the roots of the MI treatment was significantly higher than that of the PCI-5 kPa treatment by 40.3%. In addition, the ABA content in the leaves of the MI treatment was significantly higher than that of the PCI-5 kPa and PCI-15 kPa treatments by 42.1 and 30.1%, respectively; the ABA content in the bract was significantly higher than that by 40.6 and 28.2%; and the ABA content in the cob was significantly higher than that by 41.0 and 28.0%. Under 38% FC to 53% FC of SSM, the ABA content of stems and roots increased with the decrease in soil water content, and the ABA content of the PCI-5 kPa treatment was significantly lower than that of the PCI-10 kPa and PCI-15 kPa treatments. However, the ABA content in maize leaves, bracts, and cobs increased significantly at first and then decreased significantly with decreasing soil water content (Figure 4). To compare the ABA content in different organs, the average ABA content of the four treatments in each organ was calculated. The ABA content differed in different organs in the order of leaf > bract > root > cob > stem. The average ABA content in leaves was 7.4, 3.8, 2.3, and 0.3 times higher than that in bracts, roots, cobs, and stems, respectively, and that in bracts was 5.7, 2.9, and 1.7 times higher than that in roots, cobs, and stems, respectively.

#### Salicylic Acid

**FIGURE 5:**
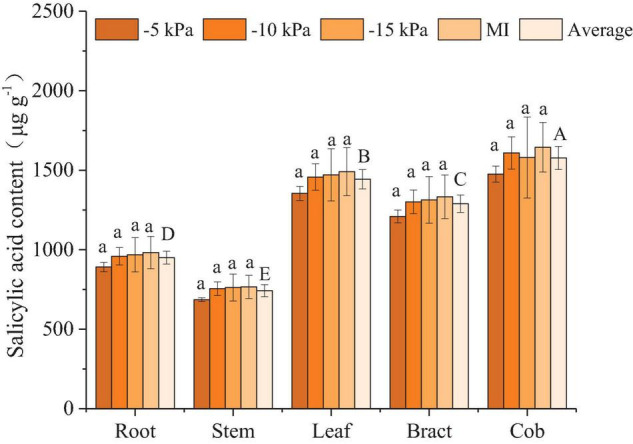
Salicylic acid content in different organs of maize under different treatments. Values represent mean ± SD. The least significant difference method was used to test differences among treatments or organs at the *P* < 0.05 level. Different lowercase letters indicate significant differences among treatments within the same organ, and different capital letters on the average value of all treatments in each organ indicate significant differences among organs.

In the present study, neither the temporal variation in soil moisture (between SSM and FSM) nor the soil water content variation under SSM had effects on the SA content of maize (Figure 5). The content of SA differed among the different organs in the order cob > leaf > bract > root > stem. The average content of SA in the cob was 112.5, 66.0, 22.3, and 9.3% higher than that in the leaf, bract, root, and stem, respectively, while that in the leaf was 94.5, 51.9, and 12.0% higher than that in the bract, root, and stem (Figure 5).

#### Root Activity and Δ ^13^C

At the grain filling stage, the root activity of maize was significantly affected by soil moisture (Table 3). Compared with MI treatment, the root activity under -5 kPa, -10 kPa, and -15 kPa of PCI increased by 57.1, 28.6, and 8.7%, respectively. The root activity of maize increased with the increase in PCI water supply pressure in the range of 38% FC to 53% FC.

**TABLE 3:** Effects of different treatments on root activity and Δ^13^C of maize.

Treatment	Root activity (mg/g/h)	Δ^13^C in maize leaf (‰)	Δ^13^C in maize bract (‰)
–5 kPa	4.56 ± 0.30^a^	5.04 ± 0.18^a^*	4.37 ± 0.07^a^
–10 kPa	3.73 ± 0.57^b^	5.06 ± 0.18^a^*	4.08 ± 0.14^b^
–15 kPa	3.15 ± 0.58^bc^	4.98 ± 0.15^a^*	4.03 ± 0.19^b^
MI	2.90 ± 0.41^c^	4.97 ± 0.18^a^*	3.70 ± 0.09^c^

*Data are the mean ± SD of the observed values, and different lowercase letters within the same column indicate significant differences among the treatments at the P < 0.05 level using the least significant difference (LSD) method.*

*Indicates a significant difference between Δ^13^C in leaves and bracts under the same treatment.

As shown in Table 3, PCI treatments had significant effects on the Δ^13^C of maize bracts but not leaves. Compared with MI treatment, the bract Δ^13^C at PCI -5 kPa, -10 kPa, and -15 kPa was significantly increased by 18.3, 10.4, and 9.1%, respectively, indicating that SSM with 53% FC, 47% FC, and 38% FC was more beneficial to the improvement of bracts Δ^13^C than FSM with 78% FC. Additionally, in the range of 38% FC to 53% FC under SSM conditions, bract Δ^13^C had an increasing trend with increasing soil water content.

### Correlation Analysis

The correlation between Δ^13^C and biomass as well as WUE was analyzed. Considering all the treatments, leaf Δ^13^C had no significant correlation with biomass, grain yield, or WUE; however, bract Δ^13^C showed a significantly positive correlation with biomass and biomass WUE. With MI treatment excluded, the above correlation remains, and the correlation coefficient between biomass and bract Δ^13^C is increased (Table 4).

**TABLE 4:** The correlation of Δ^13^C with biomass and water use efficiency.

	Dry biomass	Dry grain weight	WUE_B_	WUE_Y_
L-Δ^13^C	+0.260 (0.288)	–0.129 (–0.124)	+0.366 (+0.435)	–0.155 (–0.144)
B-Δ^13^C	+0.625** (+0.733**)	+0.312 (+0.513)	+0.788** (+0.738**)	+0.276 (+0.500)

L-Δ^13^C, leaf Δ^13^C; B-Δ^13^C, bract Δ^13^C. Values are Pearson correlation coefficients, + indicates positive correlation, – indicates a negative correlation, ** indicates significant correlation at the P < 0.01 level and the values in brackets indicate the correlation coefficient without considering MI treatment.

**FIGURE 6:**
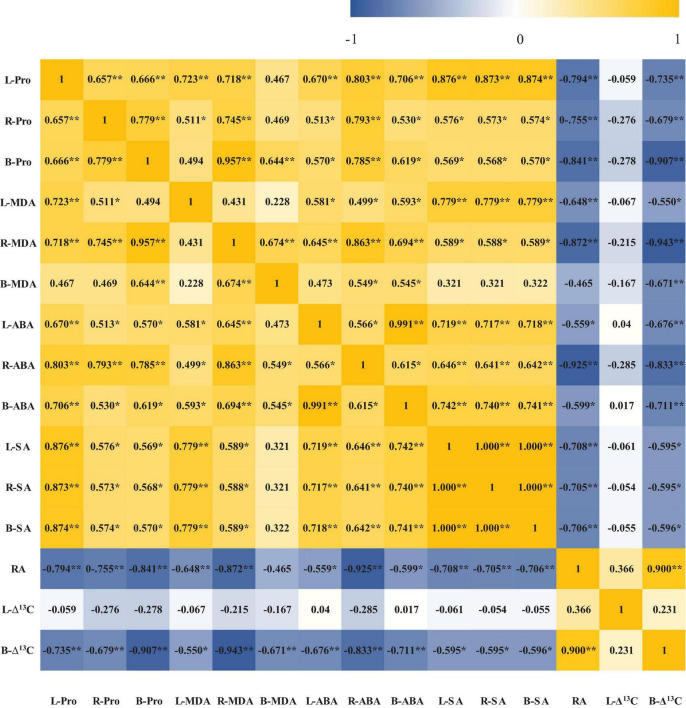
Correlation analysis of various physiological indices. L-Pro, leaf proline; R-Pro, root proline; B-Pro, bract proline; L-MDA, leaf malondialdehyde; R-MDA, root malondialdehyde; B-MDA, bract malondialdehyde; L-ABA, leaf abscisic acid; R-ABA, root abscisic acid; B-ABA, bract abscisic acid; L-SA, leaf salicylic acid; R-SA, root salicylic acid; B-SA, bract salicylic acid; RA, root activity; L-Δ^13^C, leaf Δ^13^C; B-Δ^13^C, bract Δ^13^C; *represents a significant correlation at the *P* < 0.05 level and **represents a significant correlation at the *P* < 0.05 level.

The correlations between different physiological indices were further analyzed (Figure 6), and the results indicated that root ABA was positively correlated with proline, MDA, ABA, and SA in leaves as well as proline, MDA, and SA in roots and negatively correlated with root activity and bract Δ^13^C. Moreover, root activity and bract Δ^13^C were negatively correlated with proline, MDA, ABA, and SA in leaves, roots, and bracts, and there was no significant correlation between leaf Δ^13^C and various physiological indices.

### Physiological Response of Maize Under Stable Soil Moisture

**FIGURE 7:**
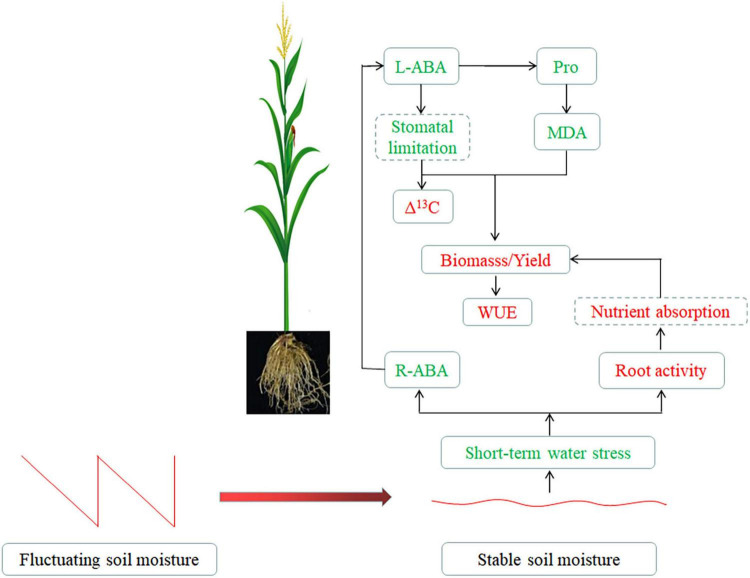
Physiological changes in maize under stable soil moisture. The green font represents the decreased indices or weakened physiological process, and the red font represents the increased index or enhanced physiological process under SSM conditions. The dotted box represents the index or physiological process not measured in this study.

The soil moisture fluctuation coefficient was smaller under SSM conditions than under FSM conditions, which reduced the short-term water stress caused by the lower soil moisture, thereby reducing the root ABA content, and improving root activity. On the one hand, due to the lower content of ABA in roots, the amount of ABA transported to the leaves was also lower. As the role of ABA was to promote stomatal closure, less ABA weakened the stomatal limitation of leaves so that the ^13^CO_2_ absorbed by the leaves was relatively low, and the Δ^13^C was relatively increased. The water stress on plants was reduced under SSM conditions, so the accumulation of the osmotic regulating substance (proline) and the membrane lipid peroxidation product (MDA) was lower, which was conducive to the improvement of yield and WUE. On the other hand, higher root activity was favorable for nutrient uptake by roots, thus improving yield and WUE (Figure 7).

## Discussion

### Physiological Responses of Maize to Temporal Variation in Soil Moisture

Plant roots cannot produce proline, and the proline in roots mainly comes from the transport of leaves (Verslues and Sharp, 1999), so the proline content in roots is the lowest (Figure 2). Previous studies suggested that the proline content of maize leaves at the grain filling stage is about 50–60 μg/g under normal water conditions, which increased to 60–100 μg/g under drought treatment (Shemi et al., 2021). The leaf proline content of PCI-5 kPa, -10 kPa, -15 kPa, and MI treatment were 48.8, 59.9, 63.6, and 67.2 μg/g, respectively. Only considering the accumulation of proline, the two treatments PCI-5 kPa and PCI-10 kPa had no water stress, and PCI-15 kPa and MI treatment caused water stress. ABA mainly existed in the leaf at the grain filling stage, and the ABA in the leaf was more sensitive to water treatment than in other organs. Additionally, the ABA was accumulated more in leaf under FSM condition than under SSM condition, which suggested that the maize was subjected to severe water stress under FSM condition than SSM (Figure 4). Previous studies under traditional irrigation conditions indicated that proline, MDA, and ABA in maize increased with the decrease of soil water content (Tayyab et al., 2020; Shemi et al., 2021), which was consistent with the changes of proline, MDA, and ABA in most organs of maize under PCI treatment. Interestingly, the average soil water contents and most organ water contents of PCI treatments were lower than that of MI treatment in this experiment (Table 2 and Supplementary Table S1), however, the proline, MDA, and ABA content in most organs of PCI treatments were lower than that of MI treatment at grain filling stage. The main reason was that the soil moistures of PCI treatments were in a stable state, while that of MI treatment was fluctuating. The stability of soil moisture under PCI can compensate for the influence of insufficient soil moisture to a certain extent, and in the case of high content of soil moisture with large temporal variation, maize would also produce adverse physiological reactions (Wang Z. et al., 2020). Previous studies had shown that under mild drought or short-term drought conditions, SA content increased slightly, but the difference was not significant (Guo et al., 2021; Mariotti et al., 2021). With the prolongation of the drought time, the SA content showed a trend of increasing first and then decreasing, and finally slightly higher than the control, but the difference was not significant (Abreu and Munné-Bosch, 2008). In the present study, the SA content of –5 kPa, –10 kPa, –15 kPa, and MI treatments also increased gradually, but the difference was not significant, indicating that MI treatment was subjected to mild or short-term drought compared with PCI treatment. The above results indicated that the proline, MDA, and ABA in most organs of maize under PCI treatments increased with the decrease of soil water content, and maize can maintain better physiological characteristics under the condition of SSM with lower water content than that of FSM.

In general, the root activity and Δ^13^C increased with the increase of soil water content. A previous study on wheat revealed that the root activity of wheat increased gradually with the soil water content increased from 40% FC to 70% FC (Wu et al., 2017). With the soil moisture measured by tensiometers, it was suggested that the Δ^13^C of the top spreading leaf of rice decreased gradually with the soil water potential changed from – 6 to – 8 kPa to – 30 to – 35 kPa (Gao et al., 2018). Only considering the same temporal variation of soil moisture in SSM, the results in the present study were consistent with the predecessors cited above, that is, in the range of 38% FC to 53% FC under SSM condition, the root activity, and bract Δ^13^C at the grain filling stage increased with the increase of soil water content. However, if all the treatments of SSM and FSM were considered, there was a contradiction between the results of the present study and that of the above literature, as bract Δ^13^C of SSM with an average moisture content of 53% FC was higher than that of FSM with an average moisture content of 78% FC. The above results indicated that the root activity and bract Δ^13^C increased with the increase of water content in the case of similar temporal variation of soil moisture. The SSM can improve the root activity and bract Δ^13^C at the grain filling stage with lower water content and make up for the negative effect brought by insufficient water content to a certain extent. The above correlation analysis revealed that ABA, osmotic regulating substances, and MDA decreased synergistically under PCI treatments, indicating that the degree of water stress and membrane lipid peroxidation under PCI treatments was slighter compared with MI treatment.

### Correlation Between Water Use Efficiency and Δ^13^C

Determination of Δ^13^C was used as a relatively convenient method to evaluate the WUE of plants under water stress (Gao et al., 2018; Yang and Li, 2018). Carbon isotope discrimination value (Δ^13^C) reflects the comprehensive information of CO_2_ fixation during photosynthesis. Because of the larger molecular weight, the diffusion rate of ^13^CO_2_ in the air is slower than that of ^12^CO_2_, and plants preferentially absorb ^12^CO_2_ when fixing CO_2_. As the photosynthesis of maize leaf is stronger than that of bract (Langdale et al., 1988), and ^12^CO_2_ is preferentially absorbed when photosynthesis is strong, so ^12^CO_2_ in bract is less than that of leaf, and Δ^13^C was smaller than that of the leaf (Table 3). Different scholars hold different views on the positive or negative correlation between Δ^13^C and biomass as well as WUE (Gao et al., 2018; Yang and Li, 2018), which may be related to the degree or duration of water stress. Underwater stress, CO_2_ assimilation is affected by a stomatal limitation or non-stomatal limitation (Sarabi et al., 2019). Under severe or long-term water stress, non-stomatal limitation occurred in leaves (Liang et al., 2019). At this time, photosynthetic activity (ATP content, Rubisco content, and activity) of mesophyll cells decreased (Hou et al., 2015), or the water loss of epidermal cells around stomata was more severe than that of guard cells, which made the stomata passively enlarged and increased intercellular carbon dioxide concentration. With the increase of intercellular carbon dioxide, the utilization ratio of ^12^CO_2_ increased, and the utilization ratio of ^13^CO_2_ decreased, resulting in increased Δ^13^C. In this case, Δ^13^C was negatively correlated with biomass and WUE. Under mild or short-term water stress, stomatal limitation occurred in leaves (Zhang Y. J. et al., 2019). In the present study, the stomatal conductivity decreased in MI treatment (Supplementary Figure S2), resulting in decreased intercellular carbon dioxide concentration. With the decreased intercellular carbon dioxide concentration, the utilization ratio of ^13^CO_2_ increases, and Δ^13^C decreases. Lower stomatal conductance may reduce photosynthesis and thus inhibited plant growth and biomass accumulation (Supplementary Figure S2). In addition, higher ABA content may promote grain abortion (Wang H. et al., 2020), resulting in lower yield and WUE (Supplementary Table S2). Therefore, the bract Δ^13^C was positively correlated with biomass and WUE based on biomass (Table 4).

Previous studies on wheat had shown that the Δ^13^C in leaves, grains, and pedicels were positively correlated with grain yield (Sayre et al., 1995; Merah et al., 1999; Tambussi et al., 2007). The results in the present study revealed that the bract Δ^13^C had positive correlations with biomass and grain yield (Table 4), which was consistent with previous research results. Studies had shown that in the case of limited water, the correlation between Δ^13^C and biomass as well as grain yield was higher than that under adequate water conditions (Gao et al., 2018), and the present study further found that the correlation between bract Δ^13^C and biomass of maize was enhanced under SSM. This may be since stomatal characteristics are in different stable states under various SSM, and gas exchange was closely related to biomass accumulation. Bracts play an important role in grain filling and yield formation of maize (Cantrell and Geadelmann, 1981). They have higher carbon assimilation efficiency than leaves to promote the accumulation of photosynthetic products (Fujita et al., 1994). Bracts produce more biomass and grain yield per unit area than leaves (Sawada et al., 1995). This may be the reason why bract Δ^13^C had a significant positive correlation with biomass and WUE based on biomass, while the Δ^13^C of leaves does not correlate with them. The results of the present study revealed that SSM enhanced the correlation between bract Δ^13^C and biomass, and bractΔ^13^C was more suitable for evaluating the WUE of maize than leaf Δ^13^C.

## Conclusion

In the present study, PCI and manual irrigation were used to form SSM and FSM with different soil moisture content in the pot experiment. The dynamic changes of soil water content from the sixth leaf stage to the maturity stage of maize under different water treatments were observed, and proline, MDA, ABA, SA, root activity, and Δ^13^C at the grain filling stage, as well as WUE throughout the growing season, were analyzed. Based on this study, the following conclusions can be drawn:

(1) SSM alleviated short-term or mild soil water stress of maize, thereby synergistically reducing the accumulation of root ABA, shoot ABA, osmotic regulating substances, and membrane lipid peroxidation products.

(2) SSM improved the root activity with even lower soil water content. The root activity of maize under the treatment of 53% FC with weak temporal variation was significantly higher than that of 78% FC with a medium temporal variation.

(3) The WUE of maize under SSM can be evaluated by bract Δ^13^C. The WUE based on biomass had a significant correlation with bract Δ^13^C at grain filling stage rather leaf Δ^13^C, and SSM enhanced the correlation between Δ^13^C and biomass. Therefore, compared with the leaf Δ^13^C, bract Δ^13^C was more suitable for evaluating WUE based on the biomass of maize.

## Data Availability Statement

The original contributions presented in the study are included in the article/Supplementary Material, further inquiries can be directed to the corresponding author/s.

## Author Contributions

LN performed the manuscript writing. LN and ZW conducted the data analysis. ZW carried out the experiment. GZ and KY contributed to perform the experiment. GL contributed to the data analysis. HL supervised the study, including design of experiments, data analysis, and manuscript writing. All the authors contributed to the article and approved the submitted version.

## Conflict of Interest

GZ was employed by Beijing Liangxiang Lanxin Hydraulic Engineering & Design Co., Ltd. The remaining authors declare that the research was conducted in the absence of any commercial or financial relationships that could be construed as a potential conflict of interest.

## Publisher’s Note

All claims expressed in this article are solely those of the authors and do not necessarily represent those of their affiliated organizations, or those of the publisher, the editors and the reviewers. Any product that may be evaluated in this article, or claim that may be made by its manufacturer, is not guaranteed or endorsed by the publisher.

## Footnotes

1
www.shimadzu.com.cn/

2
www.appliedbiosystems.com.cn/
