# Supplementary material for: Stable Soil Moisture Improves the Water Use Efficiency of Maize by Alleviating Short-Term Soil Water Stress
Source: Front Plant Sci. 2022 Apr 18;13:833041. doi: 10.3389/fpls.2022.833041 (PMC9062231; doi:10.3389/fpls.2022.833041)
Supplement: Supplementary file 1 [file Data_Sheet_1.doc]

|  | PCI-5kPa | PCI-10kPa | PCI-15kPa | MI |
| --- | --- | --- | --- | --- |
| Root | 0.78±0.03 | 0.79±0.02 | 0.74±0.02 | 0.82±0.07 |
| Shoot | 0.80±0.03 | 0.78±0.04 | 0.75±0.02 | 0.80±0.04 |
| Stem | 0.76±0.02 | 0.73±0.07 | 0.76±0.02 | 0.75±0.03 |
| Bract | 0.78±0.02 | 0.76±0.02 | 0.75±0.04 | 0.78±0.02 |
| Cob | 0.82±0.05 | 0.84±0.04 | 0.84±0.01 | 0.85±0.02 |

Table S1 Water content of different organs of maize (%).

Table S2 Effects of different treatments on water consumption, dry biomass, WUE_B_, dry grain weight and WUE_Y_ of maize (Wang et al., 2020).

| Treatment | Water consumption  (L·pot^-1^) | Dry biomass  (g) | WUE_B_  (g·kg^-1^) | Dry grain weight  (g) | WUE_Y_  (g·kg^-1^) |
| --- | --- | --- | --- | --- | --- |
| -5 kPa | 32.96±4.15 | 307.72±38.59 | 9.35±0.72 | 37.31±13.61 | 1.11±0.27 |
| -10 kPa | 29.01±2.08 | 256.03±31.53 | 8.83±0.91 | 16.91±14.77 | 0.56±0.47 |
| -15 kPa | 24.31±0.49 | 179.81±15.14 | 7.40±0.69 | 0.10±0.09 | 0.00±0.00 |
| MI | 30.48±0.21 | 221.36±16.23 | 7.26±0.53 | 18.06±3.71 | 0.59±0.12 |

Fig. S1 Dynamic changes of soil volumetric moisture content under different treatments (Wang et al., 2020).

Fig. S2 Stomatal conductance in maize leaves under different treatments.

Fig. S3 Dynamic changes of plant height(a), stem diameter(b) and leaf area(c) of maize under different treatments (Wang et al., 2020)
